# Assortative Mating in Fallow Deer Reduces the Strength of Sexual Selection

**DOI:** 10.1371/journal.pone.0018533

**Published:** 2011-04-06

**Authors:** Mary E. Farrell, Elodie Briefer, Alan G. McElligott

**Affiliations:** 1 Hartpury College, University of the West of England, Gloucester, United Kingdom; 2 School of Biological and Chemical Sciences, Queen Mary University of London, London, United Kingdom; University of Sussex, United Kingdom

## Abstract

**Background:**

Assortative mating can help explain how genetic variation for male quality is maintained even in highly polygynous species. Here, we present a longitudinal study examining how female and male ages, as well as male social dominance, affect assortative mating in fallow deer (*Dama dama*) over 10 years. Assortative mating could help explain the substantial proportion of females that do not mate with prime-aged, high ranking males, despite very high mating skew. We investigated the temporal pattern of female and male matings, and the relationship between female age and the age and dominance of their mates.

**Results:**

The peak of yearling female matings was four days later than the peak for older females. Younger females, and especially yearlings, mated with younger and lower-ranking males than older females. Similarly, young males and lower-ranking males mated with younger females than older males and higher-ranking males. Furthermore, the timing of matings by young males coincided with the peak of yearling female matings, whereas the timing of older male matings (irrespective of rank) coincided with the peak of older female matings.

**Conclusions:**

Assortative mating, through a combination of indirect and/or direct female mate choice, can help explain the persistence of genetic variation for male traits associated with reproductive success.

## Introduction

Darwin [1] distinguished two selective mechanisms in his theory of sexual selection; selection for the “*power to conquer other males in battle*” (male-male competition), or the “*power to charm the females*” (female choice). Male-male competition has received a lot more attention than either male or female choice (especially in mammals), and most studies of female mate choice have traditionally focused on male traits that are selected by the majority of females [2], [3]. Assortative mating is another important aspect of sexual selection that has been overlooked, particularly in large polygynous mammals. It occurs when individuals with certain traits or phenotypes (e.g. age, body size) mate more often with each other than is expected by chance [4], [5]. Assortative mating can have an important influence on the strength of sexual selection and can result from indirect or direct mate choice. Direct or active mate choice requires individuals to discriminate and mate more readily with certain other phenotypes [3], [6]. Indirect mate choice includes all other behaviours that limit an individual's set of potential mates [7].

According to sexual selection theory, females should actively choose high quality mates, as determined by reliable indicators of male quality [8]. Traditionally, these females were thought to benefit from increased survival, fecundity or enhanced offspring fitness [9]. However, sexual selection can fluctuate as a function of phenotypic plasticity and environmental heterogeneity [10], and therefore female mate preferences can depend on more parameters than just male quality [11]–[13]. The benefits and costs of particular choices may vary between females due to genetic and developmental differences, or even within females due to changes in the ecological or social environment that induce changes in phenotypic quality [13], [14]. Furthermore, indirect and direct mate choice can interact, such that a female's set of potential partners is reduced even before she is ready to mate.

There is evidence that female age and experience directly affect the males with which females mate. Mate choice criteria change with age in female guppies (*Poecilia reticulata* [15]), and female mate choice is dependent on size and experience in swordtail fish (*Xiphophorous multilineatus* [16]). The timing of female matings can also be affected by their age and experience, affecting their set of potential mates, and resulting in indirect mate choice. As all forms of female mate choice can significantly impact the strength and direction of sexual selection, any variation based on female age or experience may have important evolutionary implications that may alter selection for multiple male sexual traits [8], [14].

We investigated assortative mating in fallow deer (*Dama dama*) using data gathered over 10 years. It is a highly polygynous, strongly size-dimorphic and long-lived species. In our study population, the fallow bucks that gain matings usually get most of them between 5 and 8 years old [17]. Even between these ages, most matings are gained at 6–7 years old, when males are considered prime-aged [18]. Mating success is based on the number of directly observed copulations, and provides a very good estimator of reproductive success [19]. High dominance rank is strongly linked to reproductive success and there is robust evidence that reproducing males have higher phenotypic quality (i.e. higher survival rates, and more likely to mate again during subsequent breeding seasons [18], [19], [20]). The males in our study population do not lek and although establishing a territory is related to mating success, the majority of matings do not take place on territories [17], [21]. Indeed the locations of matings for males are highly unpredictable and variable, and therefore mate choice can be distinguished from female preferences for specific spatial locations [3], [21]. During the rut, many males of varying ages and ranks typically join large female groups. Furthermore, coercive matings do not occur and estrous females in our study population are highly mobile and often visit many mature males (2–9 males of 5–8 years old) before mating with one of them [22].

Female fallow deer reach sexual maturity at 18 months and can reproduce until 23 years old [23], [24]. Yearling females (one year old) are approximately 14% lighter than older females (mean: yearlings  = 37 kg; adult females  = 42 kg) and fecundity is strongly positively related to body weight in yearling females but not in older females [23]. When in oestrus, most females mate only once (84% of females [19], [22], [25]). In captivity, Komers et al. [26] found that oestrus timing was earlier when female deer were housed with 5.5 years old males compared to when they were with 2.5 years old males. This suggests that these differences might also be evident in a wild or semi-natural setting and affect the potential for assortative mating.

In order to determine the extent of assortative mating and its potential impact on the strength of sexual selection, we first assessed if the overall temporal pattern of female and male matings during the rut varied according to their ages, and according to the dominance ranks of males. We then investigated the influence of female age on the ages and dominance ranks of their mates, and vice-versa, in order to investigate if assortative mating exists in fallow deer and whether it depends on female age, and hence experience and body condition, or on male age and dominance rank. Evidence for assortative mating even in a large, highly polygynous, long-lived mammal, could suggest that this factor has been overlooked in studies of species with similar mating systems.

## Methods

### Study site and population

The study was carried out on a herd of fallow deer in Phoenix Park (709ha, 80% pasture, 20% woodland; 53° 22′ N, 6° 21′ W), Dublin, Ireland. The population size varied during the 10 year study, from 470 to 689 individuals. The majority of animals were of known age and individually recognizable, as tagging of the population by the park authorities began in 1971. Yearling females were born during June of the previous year (23 months old at time of rut) and had not mated previously. All the other females were aged 2–19 years old. For descriptive purpose, we also defined young males as males that were not socially mature (≤4 years old [27]). When males are not socially mature, they generally do not vocalise and do not actively compete for access to mating opportunities [27].

### Observations

We conducted behavioural observations during the breeding seasons from 1989–1998. We divided the breeding season into two periods. The *prerut* refers to the period when males have shed the velvet from their antlers and lasts until the day before the first mating [27]. The *rut* refers to the period during which matings occur. During the study there were seven to thirteen observers present in the field from dawn until dusk every day (approx. 11 hours) during the rut, which ensured maximum coverage of the animals. All event recording of agonistic interactions and matings (including the male and female identity) was carried out. All observers were in radio contact to facilitate the exchange of information and to prevent duplicate recording of the same behavioural events.

### Matings

We recorded 2137 matings from 1989–1998; varying from 117 matings in 1992 to 330 in 1996. Data on dominance ranks of males and on the identity of mates with which males or females were observed mating were not always available. As a result, sample sizes varied among the different analyses. Females aged between 1 and 19 years old and males aged between 3 and 9 years old were included in the analyses. All other observed age classes (females aged 20–23 years old, and males aged 1, 2 and 10 years old) were removed because of small sample sizes (*n* = 1 for each age class). Consequently, the number of matings included in our analyses was as follows: *n* = 1224 matings where female age and male rank were known; *n* = 1468 matings where female age and male age were known; *n* = 1592 matings where female age was known; *n* = 1589 matings where male rank was known and *n* = 1863 matings where male age was known.

### Dominance relationships

The outcomes of agonistic interactions recorded during the prerut (September and first half of October), were used to calculate dominance ranks for most males between 1989–1998 (one measure per male per year, except 1991 and 1992, for which rank data were not available). Male rank is thus well established before the rut so that prerut and rut rank values are highly correlated [20], [27]. The dominance rank of each male was calculated according to Clutton-Brock et al. [28] (see also [29]). We used the results of agonistic interactions (including direct and indirect wins and losses) to calculate an index of dominance as follows: Clutton-Brock Index (CBI)  =  *B* + *b* + 1/*L* + *l* + 1, where *B* is the number of males defeated by the focal male (“losers”), *b* is the number of males (excluding the focal male) defeated by the losers, *L* is the number of males that defeated the focal male (“winners”) and *l* is the number of males that defeated the winners. The male with the highest index value in each year was assigned the rank of 1 and all other males were ranked accordingly.

We calculated dominance ranks for all males that interacted with at least 10% of other males. The number of males ranked each year varied between 63 and 72 males. We considered the high-ranking males to be those with dominance ranks 1–20, the lower-ranking males to be those with dominance ranks >20, and the lowest-ranking males to be those with dominance ranks ≥40. These categories were only used for description. Analyses were carried out on continuous variables.

### Data analysis

We used Generalized Linear Mixed Models (GLMMs) fitted with Restricted Estimate Maximum Likelihood (REML, lme function in R [30]) to investigate the relationships between the following parameters: female age, male age, male dominance rank and the rut date when females and males mated. Based on scatterplots showing the relationship between the variables used in the various GLMMs, we fitted as fixed effects linear, quadratic or log terms (best fits). Because models including female and male data did not have the same random effect structure to control for repeated measurements of the same individual each year and across years (female identity nested within year of observation for females and male identity nested within year of observation for males), we decided to treat males and females separately. Because male age and male dominance rank were strongly correlated (GLMM, effect of age on rank, quadratic relationship, *n* = 1589 matings: linear term, *F*_1,1499_ = 192.16, *p*<0.0001; quadratic term, *F*_1,1499_ = 797.62, *p*<0.0001; *R^2^* = 0.87), male age and male dominance rank were also treated separately.

The first set of models assessed if the rut date when males and females were observed mating depended on female age, male age and male dominance rank. We carried out the three following models with rut date as a response variable: a) the first model investigated the effect of female age on the rut date when females were observed mating and included female age (log term) as a fixed effect and female identity nested within year of observation as a random effect; b) the second model investigated the effect of male age on the rut date when males were observed mating and included male age (linear and quadratic terms) as a fixed effect and male identity nested within year of observation as a random effect; c) the third model investigated the effect of male dominance rank on the rut date when males were observed mating and included male rank (log term) as a fixed effect and male identity nested within year of observation as a random effect. These three models also included as a covariate the total number of matings scored on each day of the rut to control for between-day variation in the availability of mating partners in our population. To allow for model comparison, we fitted these models to mating data where both female age, male age and male dominance rank were known (*n* = 1224 matings). We then compared models on the basis of the Akaike Information Criterion (AIC [31]), which is a model selection procedure well suited for observational studies, and which allows comparisons between models incorporating the same response variable but different fixed effects [32], [33]. The value of AIC for a given model is a measure of the loss of information resulting from the use of the model to explain a particular pattern (in our case, the temporal distribution of matings). Therefore, the model with the smallest AIC value is estimated to best fit the data set relative to other models considered [31]. When the difference between the AIC values of two models (*ΔAIC*) is less than 2 units, both models have support and can be considered competitive. Models with *ΔAIC* ranging from 3 to 7 are considerably less supported by the data, whereas models with *ΔAIC* >10 are poorly supported and therefore very unlikely [31].

The second set of models assessed the effect of female age on the age and dominance rank of the males they had been observed mating with. The first model included male age as a response variable and female age (linear and quadratic terms) as a fixed effect. The second model included male dominance rank as a response variable and female age (log term) as a fixed effect. In both models, female identity nested with year of observation was fitted as a random term to control for repeated measurements of the same female each year and across years.

The third set of models assessed the effect of male age and dominance rank on the age of the females they had been observed mating with. We carried out these two models with female age as a response variable. We included as a fixed effect male age (linear and quadratic terms) in the first model, and male dominance rank (linear term) in the second model. In both models, male identity nested with year of observation was fitted as a random term to control for repeated measurements of the same male each year and across years.

For each model, Q–Q plots and scatterplots of the residuals were inspected visually to ensure their normal distribution, and response variables were log-transformed when necessary. We used F-tests to assess statistical significance of the fixed effects. The calculation of a coefficient of determination *R^2^* for GLMM is not obvious because of the presence of random effects. We thus estimated *R^2^* following Magee [34] to describe the way models fitted the observed data as follows: *R^2^* = 1 – exp (- 2/*n* (*logL_M_* – *logL_0_*)), where *n* is the number of observations (matings), *logL_M_* is the standard log-likelihood of the model (which includes fixed and random effects) and *logL_0_* is the standard log-likelihood of the intercept-only model.

Additionally, the proportions of matings by yearling females versus older females, young versus older males and high-ranking versus lower-ranking males were compared using Chi-square tests (two-sided). To investigate if the timing of mating of young/older males and high-ranking/lower-ranking males was correlated with the timing of matings of yearling females or older females, we calculated, for each category, the mean number of individuals mating each day of the rut. We then confirmed the normality of the data (Kolmogorov–Smirnov test) and calculated Pearson product-moment correlations between the number of females and the number of males mating each day.

A total of 472 females and 97 different males were included in the analyses. Because 67% of females and 57% of males were present for more than one year (female presence: range = 1–9 years, mean = 2.83±0.06 years; male presence: range = 1–5 years, mean = 1.92±0.10 years), our study is partially longitudinal and allowed us to account for changes in the mate choices of individual females as they matured and aged.

Statistical analyses were carried out using R v.2.9.0 [35]. All tests were two tailed and fixed effects were considered to have a statistically significant influence if *p*<0.05. All means are given with standard errors.

## Results

### Relationship between the mating date and female age, male age and male dominance rank

**Figure 1:**
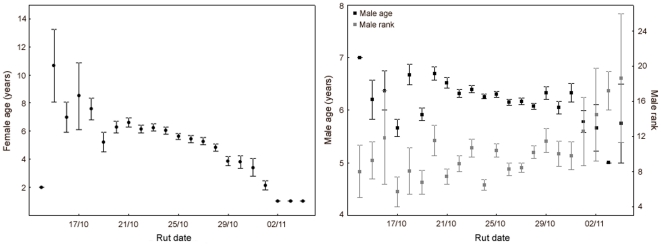
Date of matings according to female age, male age and male dominance rank. Female age (left), male age (right, black squares, left y-scale), male dominance rank (right, grey squares, right y-scale; mean±SE per year) in relation to the rut date. Lower values of dominance indicate higher-ranking males (e.g. number 1 is the top-ranked male). Younger females and males mated later in the rut than older females and males, and lower-ranking males mated later in the rut than higher-ranking ones (see Results for details).

The date of matings was influenced by both female age (GLMM, log relationship, *n* = 1224 matings: *F*_1,993_ = 139.76, *p*<0.0001; *R^2^* = 0.270), male age (GLMM, linear relationship, *n* = 1224 matings: *F*_1,1080_ = 8.64, *p* = 0.003; *R^2^* = 0.273) and male dominance rank (GLMM, log relationship, *n* = 1224 matings: *F*_1,133_ = 9.52, *p* = 0.003; *R^2^* = 0.273). Younger females (especially yearlings) and males mated later in the rut than older females and males, and lower-ranking males mated later in the rut than higher-ranking ones (Fig. 1). The AIC model selection favoured male dominance rank as the factor more strongly related to mating date (Table 1). The model incorporating male age was also well supported by the data and a close competitor to the model including male rank (*ΔAIC* = 0.94). The model including female age was less supported by the data, but also likely (*ΔAIC* = 6.65, Table 1). Thus, the variation among females in the dates of matings was partially explained by age differences, and the variation among males in the dates of matings was partially explained by differences in age and dominance rank.

**Table 1:** Relationship between the mating date and female age, male age and male dominance rank.

Date	Fixed effect	*df*	*F*	*p*	*n*	*logL_M_*	*logL_0_*	*R^2^*	*AIC*	*ΔAIC*
**a)**	Female age (log)	1,993	139.76	<.0001	1224	-2951.53	-3143.85	0.270	5948.48	6.65
**b)**	Male age	1,1080	8.64	0.003	1224	-2948.43	-3143.85	0.273	5942.77	0.94
**c)**	**Male rank (log)**	**1,133**	**9.52**	**0.003**	**1224**	**-2948.68**	**-3143.85**	**0.273**	**5941.8**	**0**

Results of the models investigating the effect of female age (log term), male age (linear term) and male dominance rank (log term) on the dates of matings. The date of matings depended on female age, male age and male dominance rank. The model selection procedure favours male age and dominance rank as the factors more strongly correlated with mating date. The model including female age is more weakly supported by the data.

The fit of the models was assessed by *R^2^* and Akaike's information criterion (*AIC*). The best model is indicated in bold. *R^2^* is calculated using the sample size (*n*), the standard log-likelihood of the model (*logL_M,_* which includes fixed and random effects) and the standard log-likelihood of the intercept-only model (*logL_0_*). *ΔAIC* gives the difference in *AIC* between each model and the best model. The three models also incorporated the number of matings each day as a covariate and individual identity (females for model a and males for models b and c) nested within year of observation.

**Figure 2:**
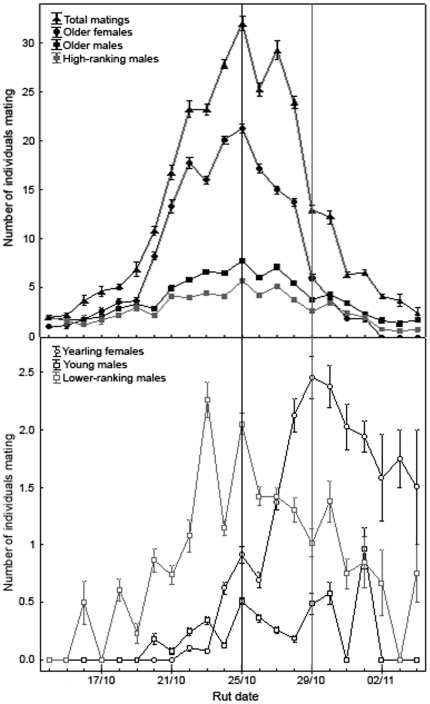
Temporal distribution of matings during the rut. Total number of matings and number of older females, older males, high-ranking males (above), yearling females, young males and lower-ranking males (below) mating on each day of the rut (mean±SE matings per year). Yearling females mated from the 22/10 until the 04/11, with a peak on 29/10 (grey line). Older females mated from the 14/10 until the 01/11 with a peak on the 25/10 (black line). The timing of matings by young males coincided with the peak of yearling female matings, whereas the timing of matings of older, high- and lower-ranking males coincided with the peak of older females mating (see Results for details).

Yearling females (<2 years old) mated from October 22 to November 04, whereas older females (2 to 19 years old) mated from October 14 to November 01 (Fig. 2). The peak of yearling female matings was 4 days later than the peak for older females (mating peak and mean mating date each year: yearling females, October 29, *n* = 128 females; older females, October 25, *n* = 1186 females; Fig. 2). Young males (3–4 years old) mated from October 20 to November 01 (mean mating date each year  =  October 27, *n* = 27 males), whereas older males (5–9 years old) mated from October 14 to November 04 (mean mating date each year  =  October 25, *n* = 162; Fig. 2). High-ranking males (ranks 1–20) mated from October 14 to November 04 (mean mating date each year  =  October 25, *n* = 93 males), whereas lower-ranking males (ranks >20) mated from October 16 to November 04 (mean mating date each year  =  October 26, *n* = 64; Fig. 2). The number of young males mating each day was positively correlated with the number of yearling females mating (Pearson product-moment correlation, *n* = 22 days: *r* = 0.44, *p* = 0.041), whereas the number of older males (*r* = 0.95, *p*<0.0001), high-ranking males (*r* = 0.90, *p*<0.0001) and lower-ranking males (*r* = 0.75, *p*<0.0001) were positively correlated with the number of older females mating (Fig. 2). The other correlations (e.g. young males and older females, older males and yearling females) were not significant (*p*>0.17 for all). To summarize, the dates of matings depended on female age, and especially on male age and on male dominance rank. Lower-ranking males, young males and yearling females mated later in the rut than, respectively, high-ranking males, older males and older females. The temporal distribution of matings by young males coincided with yearling female matings, whereas the temporal distribution of matings of both older, high-ranking and lower-ranking males coincided with older females matings.

### Influence of the age of females on the age and dominance ranks of their mates

**Figure 3:**
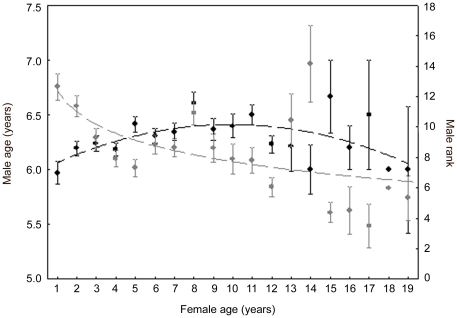
Effect of the age of mating females on the age and dominance ranks of their mates. Female age as a function of male age (black, left y-scale, quadratic relationship) and male dominance rank (grey, right y-scale, log-relationship; mean±SE). Lower values of dominance rank indicate higher-ranking males. Young and old females mated with younger males than middle-age females. Young females also mated with lower-ranking males than older females (see Results for details).

The age of females explained significantly, however weakly, the variation in the age (GLMM, quadratic relationship, *n* = 1468 matings: linear term, *F*_1,1198_ = 5.40, *p* = 0.02; quadratic term, *F*_1,1198_ = 10.28, *p* = 0.001; *R^2^* = 0.150) and the dominance rank (GLMM, log relationship, *n* = 1224 matings: *F*_1,993_ = 25.98, *p*<0.0001; *R^2^* = 0.152) of the males they mated with. Younger females (and especially yearlings) and old females (18–19 years old) mated with younger males than the other females (Fig. 3). Younger females also mated with lower-ranking males than older females (Fig. 3).

Proportionally more yearlings than older females mated with young males (yearling females: 9.9% of matings, *n* = 13 matings; older females: 1.1% of matings, *n* = 15 matings; Chi-square test: *χ* ^2^_1_ = 44.33, *p*<0.0001) and lower-ranking males (yearling females: 18.9% of matings, *n* = 20 matings; older females: 11.5% of matings, *n* = 128 matings; Chi-square test: *χ* ^2^_1_ = 4.34, *p* = 0.037; see also Fig. S1). Thus, to summarize, the variation among females in the age and dominance rank of their mates was partially explained by age differences, with yearlings mating with proportionally more young males and lower-ranking males than older females.

### Influence of the age and dominance rank of males on the age of their mates

**Figure 4:**
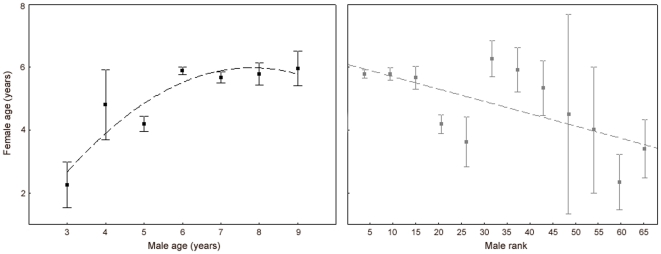
Effect of the age and dominance rank of mating males on the age of their mates. Female age as a function of male age (left) and male dominance rank (right; mean±SE female age). Lower values of dominance rank indicate higher-ranking males. Younger males and lower-ranking males mated with younger females than older and more dominant males (very weak effect: age, *R^2^* = 0.08; rank, *R^2^* = 0.07).

Both the age (GLMM, quadratic relationship, *n* = 1468 matings: linear term, *F*_1,1294_ = 19.78, *p*<0.0001; quadratic term, *F*_1,1294_ = 4.93, *p* = 0.03) and the dominance rank of males (GLMM, linear relationship, *n* = 1224 matings: *F*_1,133_ = 7.05, *p* = 0.009) explained significantly the variation in the age of females they mated with. However, the effect was very small (age, *R^2^* = 0.08; rank, *R^2^* = 0.07). Younger males and lower-ranking males mated with younger females than, respectively, older males and more dominant males (Fig. 4).

Over the 10 year period, young males (3–4 years old) gained 1.9% of matings (*n* = 35 matings), while 89.8% (*n* = 1673 matings) were gained by males between 5 and 7 years old, and 8.3% (*n* = 155 matings) were gained by older males (≥8 years old). High-ranking males (ranks 1–20) achieved 88% of matings (*n* = 1402 matings), with the top 10 ranked males overall accounting for 73.4% of matings (*n* = 1167 matings). Lower-ranking males (rank >20) gained 12% of matings (*n* = 187). Almost half of the matings (46.43%, *n* = 13 matings) gained by young males versus only 8.26% of the matings (*n* = 119 matings) by older males (5–9 years old; Chi-square test: *χ* ^2^_1_ = 44.34, *p*<0.0001) were with yearling females (see also Fig. S2). Similarly, 24.14% of matings (*n* = 7 matings) of the lowest-ranking males (ranks ≥40) versus only 7.99% (*n* = 86 matings) of matings by high-ranking males (ranks 1–20, Chi-square test: *χ* ^2^_1_ = 7.57, *p* = 0.006) were with yearling females (see also Fig. S2). Therefore, to summarize, each year, most of the matings were achieved by older and more dominant males, but a small proportion was gained by young and low-ranking males. The variation among males in the age of their mates was weakly explained by age and dominance rank differences, with young males and low-ranking males mating with proportionally more yearling females than older males and high-ranking males.

## Discussion

We investigated assortative mating in fallow deer to determine its potential impact on the strength of sexual selection in this large, highly polygynous and long-lived species. To date, many studies of large mammals with similar mating systems have focussed mainly on the attributes of highly successful males, and the very large mating skews that typically exist. The possibility that a substantial proportion of females consistently do not to mate with the “top” males has generally not been considered. Our results demonstrate that yearling fallow deer females mate later in the rut than older females, with the first yearlings not mating until eight days after the start of the rut (Fig. 3). This temporal difference in matings meant that they were more likely to mate with younger and lower-ranking males, and provides evidence for indirect mate choice [36]. Yearling females are substantially smaller than older females and the proximate factor determining the timing of mating is probably body condition. Body weight is positively related to fecundity in yearling females but not in older females [23]. Thus, age-related changes in selectivity could occur in females due to changes in body condition [23], [24], [37]. Increased mating success for younger and/or subordinate males results in weaker directional selection on male traits associated with reproductive success, such as size and social dominance [20], [38], [39], [40]. The role of this type of assortative mating in influencing sexual selection in a species such as fallow deer has been overlooked. Variation among the males that females mate with does not necessarily indicate that females have different standards of quality, but reflects the fact that yearling females in poor condition may not be able to pay the potential costs associated with mating with high quality males. These costs could include the time and energy spent mate searching, aggression from other females, or the resources required to produce offspring from the largest, most successful males [20], [41]–[43]. Alternatively, yearling females could be less experienced at discriminating between males of differing quality or at avoiding mating with young, low-ranking males [14].

We found that female age influenced the age and dominance ranks of the males they mated with; yearling females represented almost half of the matings gained by young males (46%), and they mated with more younger and lower-ranking males than older females, which mated almost exclusively with dominant males. Similarly, in an experimental study [26], older fallow deer females (>2.5 years) were found to avoid mating with younger, subordinate males, and delayed estrous even when there were costs associated with weight loss and delayed reproduction, suggesting that females choose (directly or indirectly) males according to their age. If females actively discriminate between males on the basis of age and dominance status, they could do so using information broadcast by the extremely vocal males [27]. Indeed, the vocalisations of male ungulates have been shown to contain cues to age, body size and dominance status (fallow deer [44]; red deer, *Cervus elaphus* [45], [46]; bison, *Bison bison* [47]).

Mating success and the traits associated with successful males have been well documented [3], [14], [20], but research on the matings achieved by younger and/or subordinate males in a large, polygynous mammal is extremely limited. The fallow deer is a polygynous ungulate, in which male age and dominance are highly correlated with reproductive success [19], [27]. Matings achieved by young, subordinate males are often attributed to their sneaky or coercive mating strategies [48]. However, sneaky or coercive matings are extremely rare in our study population [22], [25], and there is evidence for active female mate choice; estrous females actively avoid young males and often move between many mature males before mating with one of them [22], [25]. As the rut progresses, competition from more dominant, prime-aged males decreases as they lose condition, get challenged by subordinate males, and sometimes leave traditional rutting areas [49]–[51]. Towards the end of the rut, younger and/or subordinate males thus have increased access to estrous females, which could help explain some of the variation in the temporal mating pattern of male mating found in our study.

Assortative mating may result from lower-quality individuals mating with each other due to the inability to attract or retain a high quality mate, rather than preferences for a particular phenotype [14], [43]. Therefore, yearling females could have mated with younger and/or less dominant males due to a lack of opportunities to mate with higher quality males late in the rut. However, many older and high-ranking males were still gaining matings when yearling females started to mate (see Fig. S2), indicating that they were still available. Additionally, we have never seen males trying to avoid mating with an estrous female of any age when the opportunity arose. Female experience also plays an important role in mate selection [14]. For example, Charlton et al. [52], [53] found contrasting reactions of farm-reared red deer females in oestrus (9–15 years old) and free-ranging females (3–16 years old), to roars simulating males of sub-adult and large body sizes. Free-ranging females that were probably more experienced in terms of interacting with the males, showed greater attention to the vocalisations of sub-adult males. These sub-adult males are known to harass females, which may help explain why certain females were more attentive [53]. The assortative mating observed in our study could be explained by yearling females being less capable or experienced at discriminating between males of differing quality or at avoiding mating with young, low-ranking males [14].

When female quality varies within a population, higher quality males could preferentially mate with mature females that have higher fecundity [54], [55]. While Say et al. [19] found a strong relationship between the number of copulations observed and paternity in our study population, they also found that males whose mating success score exceeded their genetic paternity, had mated with a higher proportion of younger females (1–3 years old). These younger females may be less likely to implant a fertilized egg or maintain a developing foetus. Yearling females also give birth later (average 11 days) and to lighter offspring with higher mortality rates [23], [24], [56]. In our study, the influence of male age and male dominance rank on the age of females they mated with was very weak. Furthermore, older, high ranking males mated with young females even when there were older females still available (see Fig. S2). This suggests that male mate choice is not driving the observed assortative mating patterns.

Sexual conflict as a consequence of divergent female and male reproductive strategies plays an important role in sexual selection [57], [58], [59]. Because high quality males may sire daughters of low quality, intralocus sexual conflict [58], [60] could have important consequences for yearlings in our study population, which produce a higher proportion of female offspring than male offspring, compared with older females [37]. While females in good condition are probably able to counter the negative effects of male parental alleles [60], it is also possible that yearling females that still need resources for their own growth may gain additional benefits by not mating with high quality males. Although speculative, this suggestion is also supported by the finding that neonatal mass of fallow deer fawns is large compared to other species, and size is heritable [55], [61].

In conclusion, this study shows important differences in the temporal pattern of matings of yearling and older fallow deer females, which affect male mating success and the potential for sexual selection [3], [8]. To fully understand the fitness consequences of age-dependent female choice (both indirect and direct), an assessment of the survival and future reproductive success of offspring produced by yearling and older females, with low- or high-ranking males is required. In polygynous mating systems with very high mating skew, directional female mate choice for “good genes” should deplete the male genetic variance that is necessary for indirect genetic benefits to be maintained. This results in the evolutionary conundrum known as the “lek paradox”, one of the most important issues in studies of sexual selection [39], [62]. Our results can help explain how some of the genetic variation observed in a polygynous mammal is maintained, because a small but consistent proportion of females (mainly yearlings) do not mate with the most successful males each year. This probably results from a combination of indirect (e.g. temporal separation of estrous yearling females from some top males) and direct mate choice effects, whose overall relative contributions are difficult to assess [36].

## Supporting Information

Figure S1 **Dates of mating of yearlings and older females according to the age and dominance rank of their mates.** Number of yearlings (1 year old, above) and older females (2-19 years old, below) mating on each day of the rut with young (3-4 years old, empty squares) versus older males (5-9 years old, full squares, left) and high-ranking (ranks 1-20, empty squares) versus lower-ranking males (ranks > 20, full squares, right; mean±SE per year). The proportions of matings (%) and the total number of matings (n) with each category of males are indicated in brackets. Thus, yearling females were less selective than older females concerning the age and dominance rank of their mates throughout the rut. Older females mated almost exclusively with older and high-ranking males.
(TIF)

Figure S2 **Mating dates of young males, older males, lower-ranking males and high-ranking males according to female age.** Number of young males (3-4 years old, above left), older males (5-9 years old, below left), lower-ranking males (rank>20, above right) and high-ranking males (ranks 1-20, below right) mating on each day of the rut with yearlings (1 year old, empty circles) versus older females (2-19 years old, full circles; mean±SE per year). The proportions of matings (%) and the total number of matings (n) with each category of females are indicated in brackets. All categories of males started to mate with yearling females from the first day of their mating period (yearlings: 22/10), when older females were still mating, except lower-ranking males that started on the 27/10, when the number of older females was decreasing.
(TIF)
