# Correction: Assortative Mating in Fallow Deer Reduces the Strength of Sexual Selection

**DOI:** 10.1371/annotation/091a063b-f39b-4968-82c4-97db860ecf43

**Published:** 2011-09-23

**Authors:** Mary E. Farrell, Elodie Briefer, Tom Hayden, Alan G. McElligott

The authors would like to add Tom Hayden to the list of authors of this article. The revised byline is: Mary E. Farrell, Elodie Briefer, Tom Hayden, Alan G. McElligott. Tom Hayden's affiliation is: School of Biology and Environmental Science, University College Dublin, Belfield, Dublin, Ireland. The updated citation is: Farrell ME, Briefer E, Hayden T, McElligott AG. Assortative mating in fallow deer reduces the strength of sexual selection. PLoS One. 2011 Apr 6;6(4):e18533.

Prof Hayden established the research program that led to the collection of this dataset and has actively participated in the collection of data since 1988. Prof Hayden has no competing interests.

