# Supplementary figures and images for: Assortative Mating in Fallow Deer Reduces the Strength of Sexual Selection
Source: PLoS One. 2011 Apr 6;6(4):e18533. doi: 10.1371/journal.pone.0018533 (PMC3071821; doi:10.1371/journal.pone.0018533)

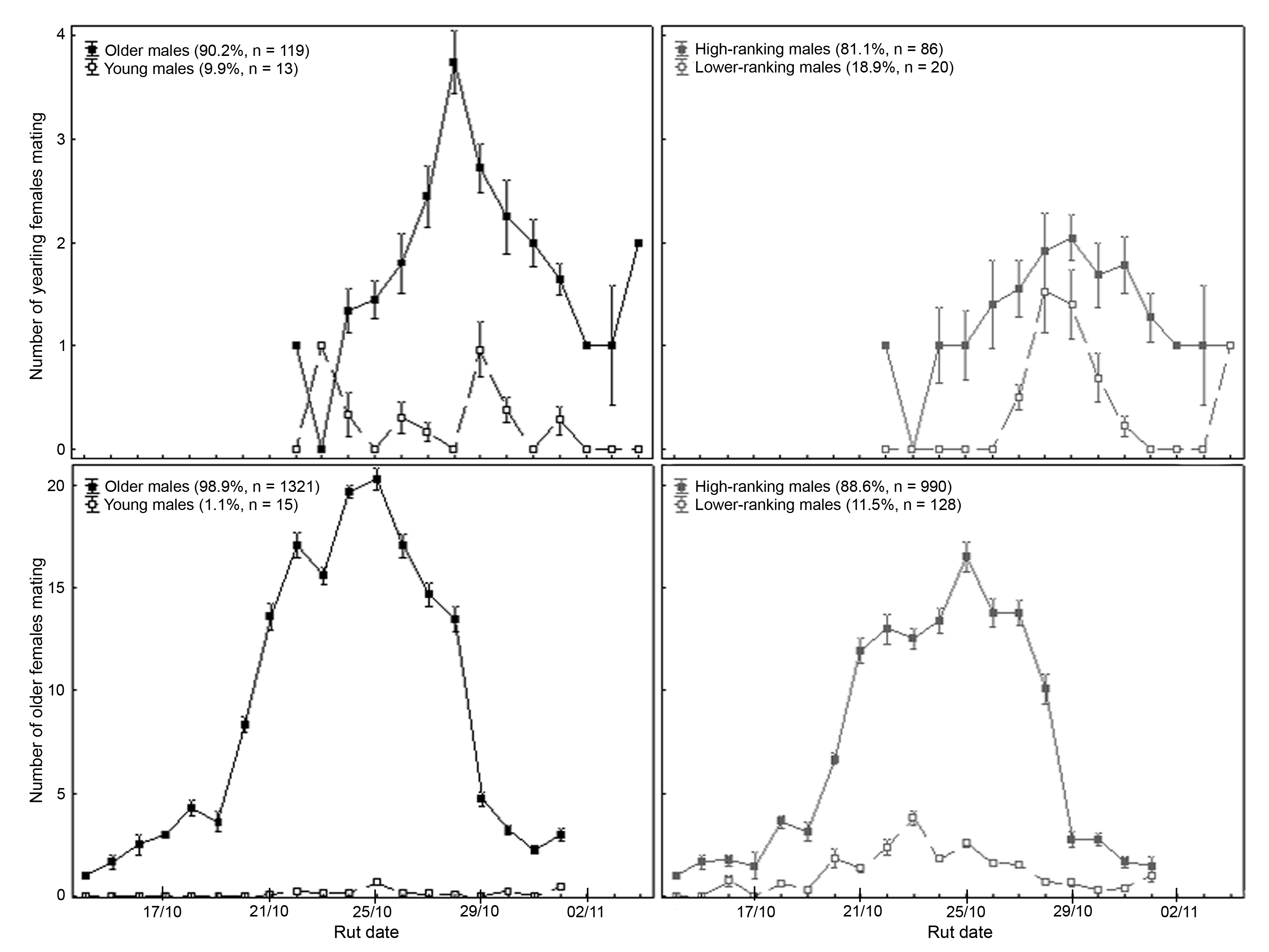

Supplement: Figure S1 — Dates of mating of yearlings and older females according to the age and dominance rank of their mates. Number of yearlings (1 year old, above) and older females (2-19 years old, below) mating on each day of the rut with young (3-4 years old, empty squares) versus older males (5-9 years old, full squares, left) and high-ranking (ranks 1-20, empty squares) versus lower-ranking males (ranks > 20, full squares, right; mean±SE per year). The proportions of matings (%) and the total number of matings (n) with each category of males are indicated in brackets. Thus, yearling females were less selective than older females concerning the age and dominance rank of their mates throughout the rut. Older females mated almost exclusively with older and high-ranking males. (TIF) [file pone.0018533.s001.tif]

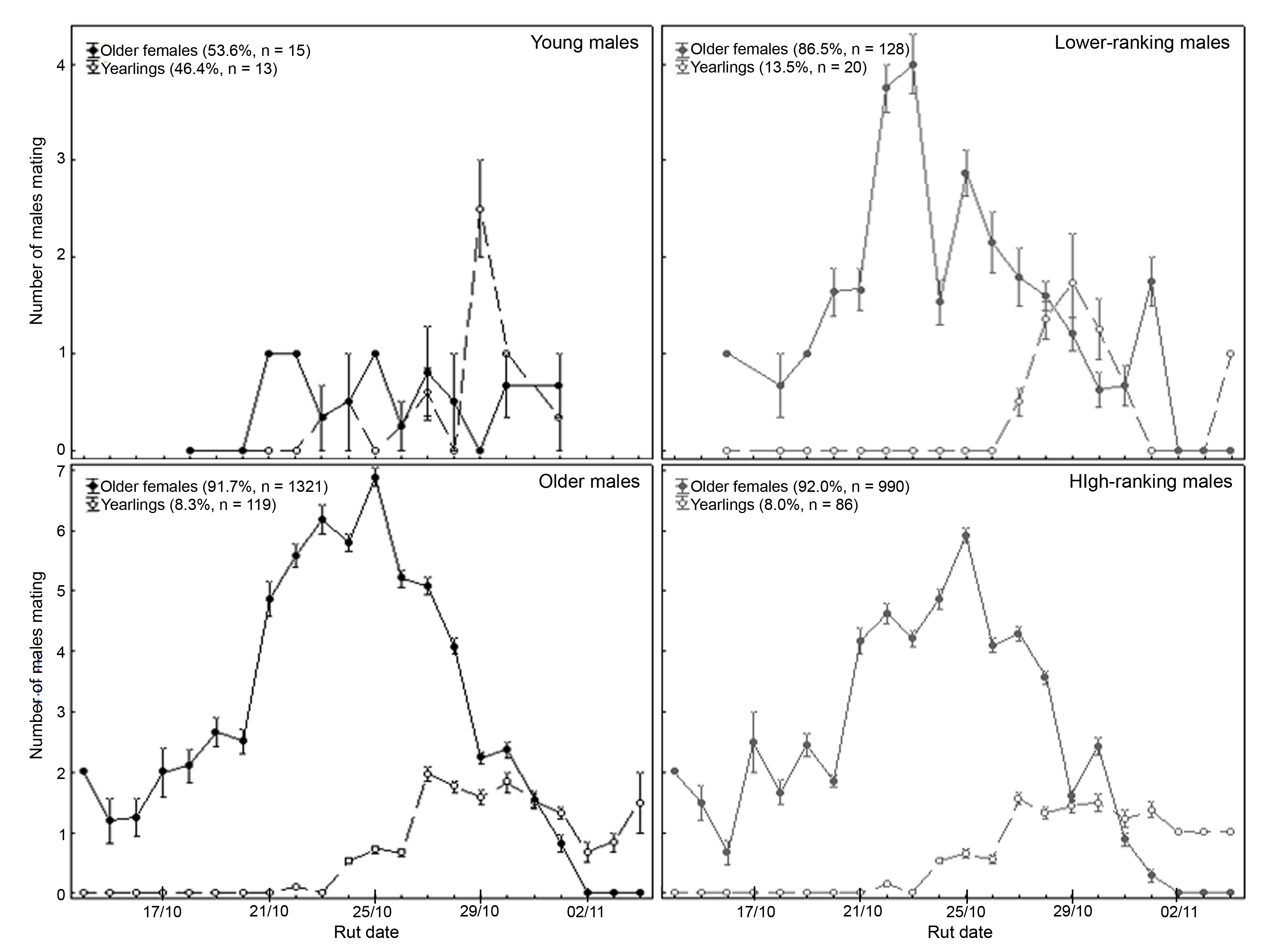

Supplement: Figure S2 — Mating dates of young males, older males, lower-ranking males and high-ranking males according to female age. Number of young males (3-4 years old, above left), older males (5-9 years old, below left), lower-ranking males (rank>20, above right) and high-ranking males (ranks 1-20, below right) mating on each day of the rut with yearlings (1 year old, empty circles) versus older females (2-19 years old, full circles; mean±SE per year). The proportions of matings (%) and the total number of matings (n) with each category of females are indicated in brackets. All categories of males started to mate with yearling females from the first day of their mating period (yearlings: 22/10), when older females were still mating, except lower-ranking males that started on the 27/10, when the number of older females was decreasing. (TIF) [file pone.0018533.s002.tif]
